# A genetic study on *C5-TRAF1* and progression of joint damage in rheumatoid arthritis

**DOI:** 10.1186/s13075-014-0514-0

**Published:** 2015-01-08

**Authors:** Hanna W van Steenbergen, Luis Rodríguez-Rodríguez, Ewa Berglin, Alexandra Zhernakova, Rachel Knevel, Jose Ivorra-Cortés, Tom WJ Huizinga, Benjamin Fernández-Gutiérrez, Peter K Gregersen, Solbritt Rantapää-Dahlqvist, Annette HM van der Helm-van Mil

**Affiliations:** Department of Rheumatology, Leiden University Medical Center, P.O. Box 9600, Leiden, 2300 RC the Netherlands; Department of Rheumatology and Health Research Institute, San Carlos Clinical Hospital, Madrid, Spain; Department of Public Health and Clinical Medicine/Rheumatology, University Hospital, Umeå, Sweden; Department of Genetics, University Medical Center Groningen, Groningen, the Netherlands; Department of Rheumatology, University Hospital la Fe, Valencia, Spain; Feinstein Institute for Medical Research and North Shore-LIJ Health System, Manhasset, NY USA

## Abstract

**Introduction:**

The severity of joint damage progression in rheumatoid arthritis (RA) is heritable. Several genetic variants have been identified, but together explain only part of the total genetic effect. Variants in *Interleukin-6 (IL-6), Interleukin-10 (IL-10), C5-TRAF1,* and *Fc-receptor-like-3 (FCRL3)* have been described to associate with radiographic progression, but results of different studies were incongruent. We aimed to clarify associations of these variants with radiographic progression by evaluating six independent cohorts.

**Methods:**

In total 5,895 sets of radiographs of 2,493 RA-patients included in six different independent datasets from the Netherlands, Sweden, Spain and North-America were studied in relation to rs1800795 *(IL-6)*, rs1800896 *(IL-10),* rs2900180 (*C5-TRAF1)* and rs7528684 *(FCRL3)*. Associations were tested in the total RA-populations and in anti-citrullinated peptide antibodies (ACPA)-positive and ACPA-negative subgroups per cohort, followed by meta-analyses. Furthermore, the associated region *C5-TRAF1* was fine-mapped in the ACPA-negative Dutch RA-patients.

**Results:**

No associations were found for rs1800795 *(IL-6)*, rs1800896 *(IL-10)* and rs7528684 *(FCRL3)* in the total RA-population and after stratification for ACPA. Rs2900180 in *C5-TRAF1* was associated with radiographic progression in the ACPA-negative population (*P*-value meta-analysis = 5.85 × 10^−7^); the minor allele was associated with more radiographic progression. Fine-mapping revealed a region of 66Kb that was associated; the lowest *P*-value was for rs7021880 in *TRAF1*. The *P*-value for rs7021880 in meta-analysis was 6.35 × 10^−8^. Previous studies indicate that the region of rs7021880 was associated with RNA expression of *TRAF1* and *C5*.

**Conclusion:**

Variants in *IL-6, IL-10* and *FCRL3* were not associated with radiographic progression. Rs2900180 in *C5-TRAF1* and linked variants in a 66Kb region were associated with radiographic progression in ACPA-negative RA.

**Electronic supplementary material:**

The online version of this article (doi:10.1186/s13075-014-0514-0) contains supplementary material, which is available to authorized users.

## Introduction

Thanks to the introduction of novel treatments and up-to-date treatment strategies, the severity of joint destruction in rheumatoid arthritis (RA) has decreased considerably [1]. Nonetheless, in daily clinical practice radiographic progression is still prevalent and understanding the mechanisms underlying the inter-individual differences in radiographic progression is relevant. The heritability of joint destruction has been estimated to be 45% to 58% [2]. Thus far, several genetic risk factors for radiographic progression have been replicated in independent studies or found significant in meta-analyses of different cohorts, but together explain only 18% of variance in radiographic progression [3].

Part of the ‘missing heritability’ might be explained by not yet identified common genetic variants that associate with radiographic progression in RA. The literature on genetic variants for radiographic progression was reviewed recently [4]. Published and yet unpublished data were combined, and it was concluded that for 12 genetic variants their associations with radiographic progression were either replicated in independent cohort studies or found significant in meta-analysis of multiple cohorts. However, the associations between rs1800795 in *Interleukin (IL)-6*, rs1800896 in *IL-10*, rs2900180 in *C5-TRAF1* and rs7528684 in *Fc-receptor-like-3 (FCRL3)* and joint damage were not clear [4].

Rs1800795 in *IL-6* was associated with radiographic joint damage at baseline in 964 United Kingdom (UK) RA patients, but the association was mainly observed in autoantibody-positive patients [5]. *IL-10* was observed as a severity factor evaluating 138 RA patients [6], but not in a study of 108 RA patients [7]. Rs2900180 in *C5-TRAF1* was identified in a cross-sectional study [8]; it was also found significant in another UK cohort [9], but not in other datasets [4]. Rs7528684 in *FCRL3* was observed as a severity factor in two studies [10,11], although the association was once restricted to the subgroup with a disease duration of at least 10 years [11] and not found in other datasets [4,12].

Presumably, the scarcity of large well-defined longitudinal cohorts of RA patients who were treated in eras when early, tailored treatment and use of biologics were uncommon may have contributed to the incongruent findings.

In order to increase the comprehension on the associations of these variants with radiographic progression in RA and in the anti-citrullinated peptide antibodies (ACPA)-positive and ACPA-negative subgroups, we performed the present study and evaluated these genetic variants in six independent European and North American RA cohorts in one of the largest studies to date on RA severity.

## Methods

### Study population

The six cohorts consisted in total of 5,895 sets of radiographs of 2,493 RA patients who fulfilled the 1987 American College of Rheumatology (ACR) criteria (Table 1). All patients gave their informed consent and approval was obtained from the local Ethical Committee of each hospital (METC Leiden University Medical Center, EPN University Hospital Umeå, San Carlos Clinical Hospital Ethics Committee, Via Christi IRB and North Shore-LIJ Health System IRB).

**Table 1:** **Patient characteristics**

	**Leiden EAC**	**Umeå**	**HCSC-RAC**	**Wichita**	**NDB**	**NARAC**	**Total**
Total number of patients	597	459	383	101	568	385	2,493
Total number of sets of radiographs	3,143	868	573	358	568	385	5,895
Radiographic follow-up in years^a^	7	2	10	15	NA	NA	
Disease duration in years at radiograph, mean (SD)^b^	NA	NA	NA	NA	10.1 (5.1)	13.9 (10.5)	
Method of scoring	SHS	Larsen	SHS	SHS	SHS	SHS	
Year of diagnosis	1993-2006	1995-2010	1976-2011	1963-1999	1980-1999	1953-2002	
Female, number (%)	402 (67.3)	321 (69.9)	293 (76.5)	70 (69.3)	444 (78.2)	281 (73.0)	
Age at diagnosis in years, mean (SD)	57.1 (15.6)	53.9 (14.5)	47.0 (14.0)	49.0 (11.7)	48.6 (12.7)	40.8 (11.9)	
ACPA-positive, number (%)	309 (52.8)^c^	339 (73.9)	165 (49.3)^d^	97 (96.0)^e^	453 (79.8)	385 (100)	
MAF rs1800795 (G) (*IL-6*), %	42.0	46.5	33.8	46.5	40.8	42.1^f^	
MAF rs1800896 (T) (*IL-10*), %	48.1	44.3	52.7	49.0	48.5	45.6	
MAF rs2900180 (A) (*C5-TRAF1*), %	36.0	36.4	27.8	36.6	35.6	39.0	
MAF rs3761959 (A) (*FCRL3*), %^g^	45.5	44.2	42.2	50.0	49.7	47.4	

^a^For the studies with longitudinal radiographic data (more than one radiograph in time), the maximum radiographic follow-up duration was reported; ^b^for the studies with one radiograph per patient, the mean disease duration at time of the radiograph was reported; ^c^ACPA status missing in 12 patients from the Leiden EAC cohort; ^d^ACPA status missing in 48 patients from the HCSC-RAC cohort; ^e^ACPA status missing in one patient from the Wichita cohort; ^f^Data on rs1800795 were not available in the NARAC; data on a proxy rs1554606 (R^2^ = 0.868) were available; ^g^in all cohorts data on rs7528684 were not available; data on a perfect proxy rs3761959 (R^2^ = 1.000) were available. ACPA, anti-citrullinated peptide antibodies; EAC, Early Arthritis Clinic; HCSC-RAC, Hospital Clinico San Carlos – Rheumatoid Arthritis Cohort; MAF, minor allele frequency; NA, not applicable; NARAC, North American Rheumatoid Arthritis Consortium; NDB, National Data Bank for Rheumatic Diseases; SD, standard deviation; SHS, Sharp-van der Heijde score.

#### Leiden Early Arthritis Clinic (EAC)

This cohort contained 597 Dutch early RA patients included between 1993 and 2006 [13]. At baseline and during yearly follow-up visits over seven years, 3,143 sets of hand and feet radiographs were made and chronologically scored by one experienced reader according to the Sharp-van der Heijde method (SHS) (within reader intraclass correlation coefficients (ICC) 0.91). The initial treatment strategy differed for different inclusion periods: patients included in 1993 to 1995 were initially treated with non-steroidal anti-inflammatory drugs (NSAIDs), patients included in 1996 to 1998 were initially treated with hydroxychloroquine or sulfasalazine and patients included in 1999 to 2006 were promptly treated with methotrexate [13].

#### Umeå

This cohort involved 459 Swedish early RA patients included between 1995 and 2010. At baseline and after two years in total, 868 radiographs of hands and feet were made and scored using the Larsen score by two trained readers as described previously [14]. Treatment strategies differed between 1995 and 2000, 2000 and 2005 and 2006 and 2010, resulting in less severe radiographic progression in the subsequent treatment periods.

#### Hospital Clinico San Carlos – rheumatoid arthritis cohort (HCSC-RAC)

This Spanish cohort comprised 383 early RA patients, diagnosed between 1976 and 2011 [15]. During the first 10 years after disease-onset 573 radiographs of hands were made and scored chronologically according to the SHS (ICC 0.99). Initial treatment strategies differed for different inclusion periods: <1990 (initial treatment with NSAIDs), 1990 to 1999 (initial monotherapy conventional disease-modifying antirheumatic drugs (DMARDs), 2000 to 2004 (initial monotherapy regularly and combination therapy rarely), 2005 to 2009 (initial combination therapy regularly used as well as biologics) and 2010 to 2011 (tailored treatment).

#### Wichita

This cohort comprised 101 patients from one practice in Wichita (KS, US) diagnosed between 1963 and 1999 [16]. In total, 358 sets of hand radiographs were made during the first 15 years after disease onset and scored with known time-order using the SHS (ICC 0.98).

#### National data bank for rheumatic diseases (NDB)

This dataset included 568 patients from the US and Canada, who were diagnosed between 1980 and 1999 [17]. One radiograph set of the hands was available per patient and SHS-scored (ICC 0.98).

#### North American Rheumatoid Arthritis Consortium (NARAC)

This dataset comprised 385 unrelated RA patients, who were diagnosed between 1953 and 2002 [18]. One radiograph set of the hands was available per patient. The radiographs were SHS-scored (ICC 0.99). The patients in the three North American cohorts developed RA in eras when early, tailored treatment and use of biologics were uncommon; no treatment effects were observed for different era of diagnoses.

### Genotyping

In the EAC, Umeå, HCSC-RAC, Wichita and NDB cohorts genotyping was done using the Immunochip according to Illumina’s protocols as described previously [19,20]. In the NARAC genotyping was performed using the Illumina Hapmap 500 BeadChip as described elsewhere [18]. Genotyping data were extracted of rs1800795 in *IL-6*, rs1800896 in *IL-10*, rs2900180 in *C5-TRAF1* and rs7528684 in *FCRL3*. Data on rs1800795 were not available in the NARAC but genotyping data of a proxy rs1554606 (R^2^ = 0.868 and D’ = 0.932) were retrieved. In all cohorts, data on rs7528684 (*FCRL3*) were not available; data on a perfect proxy rs3761959 (R^2^ and D’ both 1.000) were studied.

### Fine-mapping

The *C5-TRAF1* region was fine-mapped in ACPA-negative patients of the EAC. Data of genetic variants in the region of rs2900180 were retrieved using the Immunochip, starting at the upstream haplotype block of *PHF19* until the downstream haplotype block of *C5* (chromosome 9: 122,680 Kb to 122,927 Kb). Genotypic data were accepted after quality control as described elsewhere [20], requiring minor allele frequency (MAF) >0.0001, Hardy-Weinberg equilibrium (HWE) *P* >0.001 and genotyping success rate >0.99. Genetic outliers and relatives (both defined by principal component analysis) and patients with a gender mismatch between the data file and DNA were excluded. In this way, 424 SNPs were obtained and analyzed for their association with radiographic progression. The variant with the strongest association was subsequently associated with radiographic progression in the ACPA-negative patients of the Umeå, HCSC-RAC and NDB cohorts.

### Downstream effect

To identify functional downstream effects of *C5-TRAF1*, a search was performed in publically available databases and datasets [21-26]. Explored were the RegulomeDB [21], datasets that have evaluated constitutive RNA expression by mapping expression quantitative trait locus (eQTL) in peripheral blood samples from 8,086 individuals [22] and purified CD4+ T-cells and monocytes from 461 individuals [23], and datasets that have evaluated response eQTLs (QTLs associated with change in expression after stimulation) on lymphoblastoid cell lines from 40 individuals [24], monocytes from 432 individuals [25] and monocytes derived dendritic cells from 534 individuals [26].

### Statistical analysis

Associations between genotypes and radiographic joint damage were analyzed per cohort using an additive model. In all datasets, radiographic scores were log-transformed (log10(radiographic score +1)) to approximate a normal distribution. The residuals of the used models were normally distributed around the zero-line in all cohorts, indicating a good fit of the models (Additional file 1).

In the cohorts with multiple sets of radiographs over time (EAC, Umeå, HCSC-RAC and Wichita) a multivariate normal regression model for longitudinal data was used with radiographic scores as response variable. This method takes advantage of the within-person correlation between repeated measurements; as such, the radiographic progression rates were estimated more precisely in the cohorts with serial radiographs compared to datasets with one radiograph per patient (for a detailed description see reference [27]). The obtained effect size (beta) was back-transformed to the normal score and indicated the fold rate of joint destruction per year per minor allele compared to the reference genotype.

In the cohorts with a set of radiographs at one time-point (NDB and NARAC) the estimated yearly progression rate was calculated (total SHS divided by number of disease year at the time of the radiograph) in order to make the estimates of the progression rates comparable to those in the other datasets. A linear regression analysis was used with estimated yearly progression as outcome variable. Here, also, the obtained effect size was back-transformed and indicated the fold rate of joint destruction per year per minor allele compared to the reference common genotype.

In all datasets, adjustments were made for age and gender. In the cohorts that included patients in periods with different treatment strategies (EAC, Umeå and HCSC-RAC) analyses were also adjusted for the inclusion period as proxies for differences in treatment strategies.

The majority of datasets studied were estimated to be insufficiently powered to find statistically significant associations in the individual cohorts. Therefore, the effect sizes and standard errors of the individual analyses were combined in an inverse-weighted variance meta-analysis to test the overall association. This was allowed because the obtained effect sizes of the individual datasets, although different methods were used to score joint destruction (SHS and Larsen), all represented the relative increase (without units) of progression in joint destruction per year. The meta-analysis weights the results with a low standard error stronger than the results with a high standard error, preventing an overrepresentation of less precise data on the outcome. Subsequently, datasets with smaller 95% confidence intervals (CI) had a larger weight in the meta-analysis.

The cut-off for statistical significance was set at *P* <4.17 × 10^−3^ using the Bonferroni correction (four variants tested in the total RA population and ACPA-positive and ACPA-negative subgroups: 0.05/12 tests = 4.17 × 10^−3^). For the fine-mapping analyses the cut-off for statistical significance was set at *P* <1.18 × 10^−4^, also using the Bonferroni correction (0.05/424 tests = 1.18 × 10^−4^). Analyses were performed using IBM SPSS version 20 and Stata version 12.0.

## Results

### Patient characteristics and analyses on total RA-population

**Figure 1:**
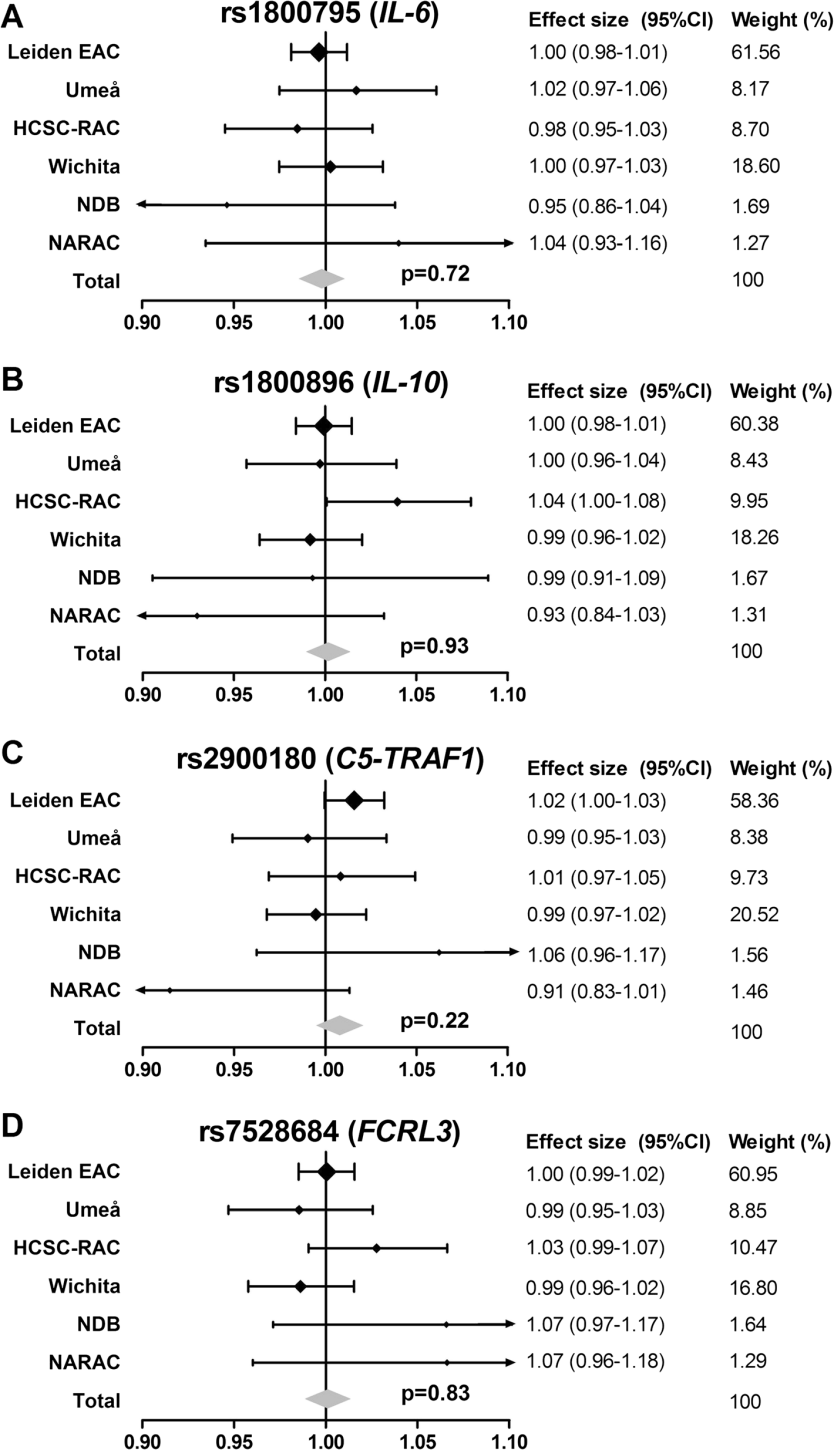
**Genetic variants in** ***IL-6*** **(A),** ***IL-10*** **(B),** ***C5-TRAF1*** **(C) and** ***FCRL3*** **(D) in relation to radiographic joint damage progression.** Presented are the yearly radiographic progression rates per individual cohort and the meta-analyses evaluating the six cohorts combined, consisting in total of 2,493 patients and 5,895 sets of radiographs. None of the studied genetic variants were significantly associated with radiographic progression, neither in the individual cohorts nor in meta-analysis. Rs1800795 (*IL-6*) I^2^ 0.0%, *P* 0.67; *P* fixed effect 0.72, *P* random effect 0.72; rs1800896 (*IL-10*) I^2^ 20.8%, *P* 0.28; *P* fixed effect 0.93, *P* random effect 0.89; rs2900180 (*C5-TRAF1*) I^2^ 28.7%, *P* 0.22; *P* fixed effect 0.22, *P* random effect 0.63; rs7528684 (*FCRL3)* I^2^ 26.0%, *P* 0.24; *P* fixed effect 0.83, *P* random effect 0.73.

The minor allele frequencies for rs1800795 (G) in *IL-6*, rs1800896 (T) in *IL-10*, rs2900180 (A) in *C5-TRAF1* and s3761959 (A) (=perfect proxy rs7528684 (G) in *FCRL3* in the different cohorts are presented in Table 1. First, analyses were done on the total RA-population. Statistical significance was obtained in none of the individual cohorts. The directionality of the effects was variable across the cohorts (Figure 1). Also in the meta-analyses on the six cohorts (2,493 patients and 5,895 sets of radiographs in total) no significant associations were obtained for rs1800795 in *IL-6* (fixed effects model *P* = 0.72), rs1800896 in *IL-10* (fixed effects model *P* = 0.93), rs2900180 in *C5-TRAF1* (fixed effects model *P* = 0.22) and rs7528684 in *FCRL3* (fixed effects model *P* = 0.83).

### Analyses of ACPA-positive and ACPA-negative rheumatoid arthritis

As some of the initial reports on these four genetic variants stratified or adjusted the analyses for the presence of ACPA and as ACPA-positive and ACPA-negative RA are considered as separate disease entities, analyses were performed on radiographic progression in ACPA-positive and ACPA-negative RA separately. The ACPA-positive subgroup comprised 1,748 patients (with 3,820 sets of radiographs) who were included in six cohorts. The ACPA-negative subgroup included 681 patients (with 1,933 sets of radiographs) who were included in the EAC, Umeå, HCSC-RAC and NDB cohorts (Table 1).

**Figure 2:**
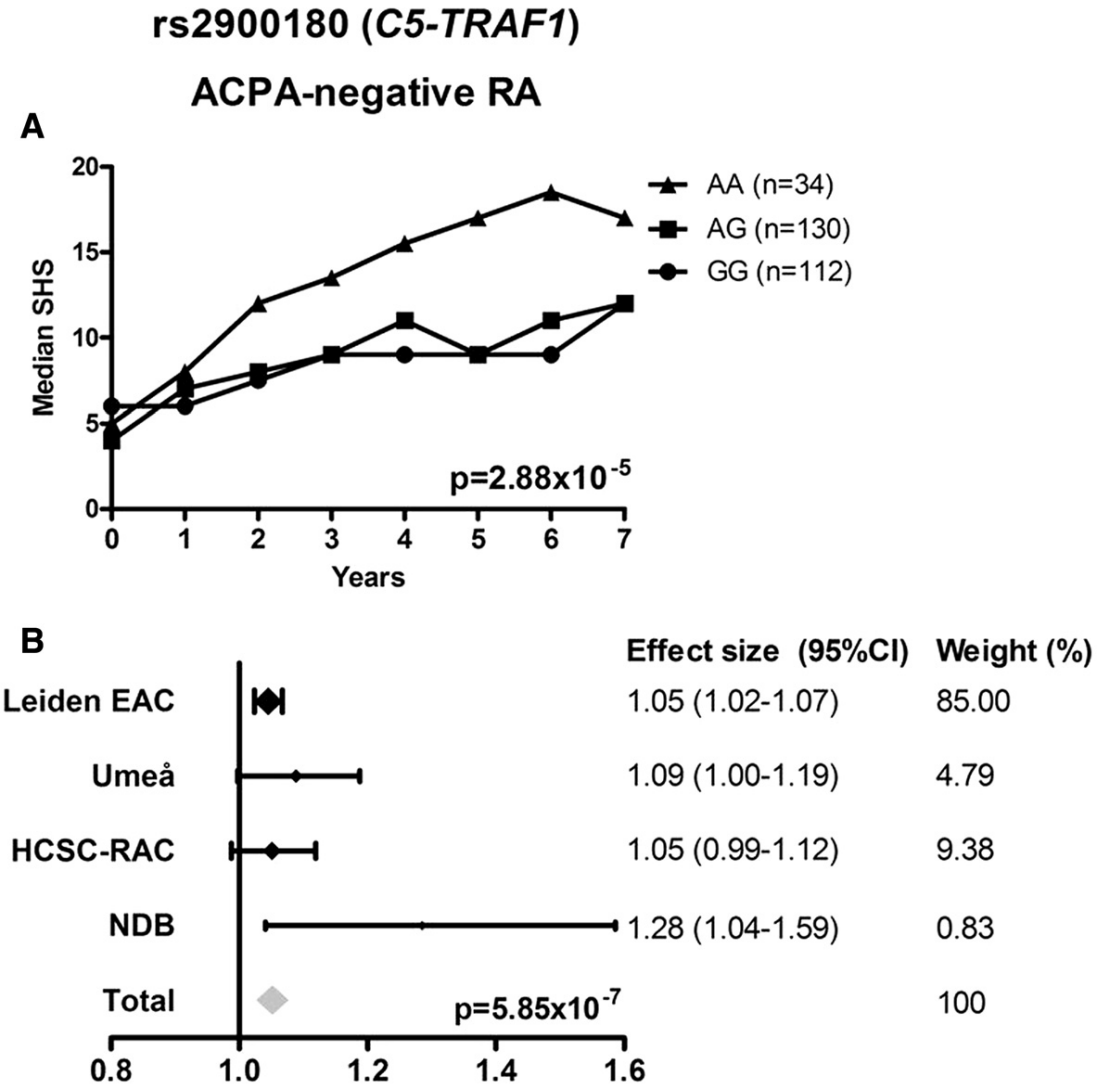
**Rs2900180 in** ***C5-TRAF1*** **in relation to radiographic progression in ACPA-negative RA patients. (A)** Depicted are the median SHSs during seven years of follow-up of ACPA-negative RA patients with different genotypes in the Leiden EAC. Patients had per minor allele a 1.045 fold rate of joint destruction per year compared to patients with the common genotype (*P* = 2.88 × 10^−5^). **(B)** Yearly radiographic progression rates per individual cohort and the meta-analysis evaluating the cohorts with ACPA-negative patients. Analysis of the ACPA-negative subgroup of the Wichita cohort was not performed as it included only three ACPA-negative patients. I^2^ 33.0%, *P* = 0.22; *P* fixed effect = 5.85 × 10^−7^, *P* random effect = 0.0024.

Rs1800795 (*IL-6*), rs1800896 (*IL-10*), and rs7528684 (*FCRL3*) were not associated with radiographic progression, neither in the ACPA-positive nor in the ACPA-negative group of RA-patients (Additional file 2). Rs2900180 in *C5-TRAF1* was not associated with radiographic progression in ACPA-positive RA (Additional file 2). In contrast, in ACPA-negative RA a significant association with radiographic progression was observed in the EAC (*P* = 2.88 × 10^−5^) (Figure 2A). The directionality of the effect was similar in the Umeå, HCSC-RAC and NDB cohorts. Also, the meta-analysis revealed a significant association (fixed effects model *P* = 5.85 × 10^−7^) (Figure 2B). In all cohorts, patients with the minor allele had a higher rate of joint destruction. For instance, RA patients included in the EAC with one minor allele had a 1.045 fold rate of joint destruction per year compared to patients with the common genotype; this equals a 36% (1.045^7) higher rate of joint destruction over seven years (Figure 2A).

### Fine-mapping

**Figure 3:**
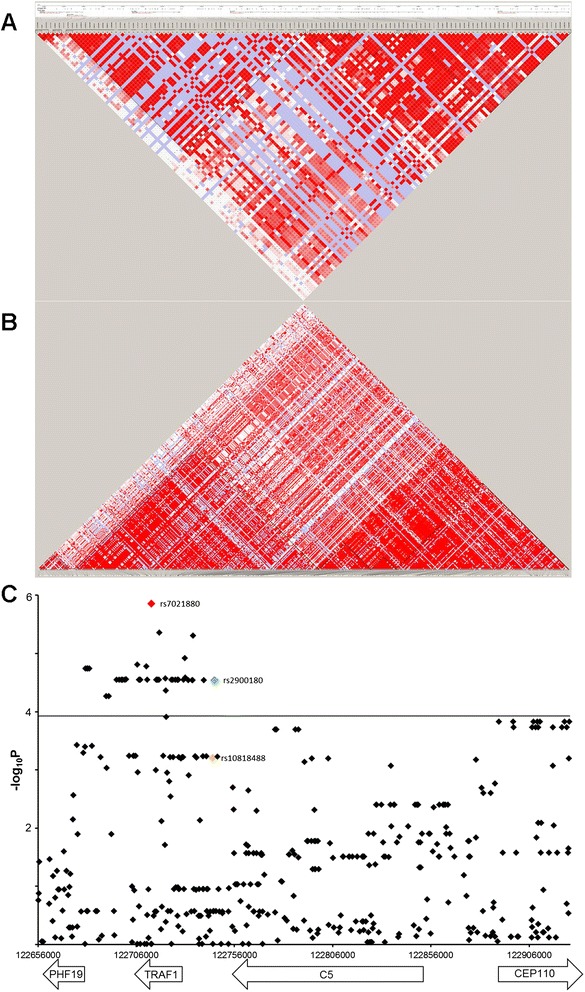
**LD plots of the** ***C5-TRAF1*** **region. (A)** Total region in hapmap CEU patients and **(B)** fine-mapped in Leiden EAC. The colors reflect de D’ between the SNPs. Coordinates relate to NCBI36 hg18 release 2006. **(C)** Results of the multivariate normal regression analysis for 424 variants in the *C5-TRAF1* region in the ACPA-negative patients of the EAC. Rs2900180 is the initially studied variant (green), rs7021880 is the variant with the lowest *P*-value (red) and rs10818488 is the variant we previously studied in relation to radiographic progression (yellow) and did not associate with radiographic progression in the total population [29]. Also in the current study, rs10818488 did not pass the cut-off for multiple testing correction in the ACPA-negative patients. Using the Bonferroni correction (considering 424 variants studied) the cut-off for statistical significance was set at 1.18 × 10^−4^ as represented by the horizontal line. ACPA, anti-citrullinated peptide antibodies; EAC, Early Arthritis Clinic; LD, linkage disequilibrium.

To examine if other genetic variants within the *C5-TRAF1* region had statistically stronger associations with the rate of joint destruction than rs2900180, this region was fine-mapped in the ACPA-negative EAC patients. In total, 43 variants had a *P*-value below the threshold for multiple correction (*P* <1.18 × 10^−4^) of which 34 were statistically more strongly associated with radiographic progression than rs2900180 (Figure 3, Additional file 3). The 43 associating variants, including rs2900180, were all located within a 66 Kb region spanning *TRAF1* and extending downstream to the *C5-TRAF1* intergenic region and upstream to the *TRAF1-PHF19* intergenic region. The variant with the lowest *P*-value was rs7021880 located in *TRAF1* (beta = 1.052 per year per minor allele, *P* = 1.39 × 10^−6^). In a conditional analysis on rs2900180 and rs7021880 (R^2^ = 0.864), both variants lost statistical significance (rs2900180 beta = 0.99 *P* = 0.77; rs7021880 beta = 1.06 *P* = 0.057). This suggests that these two variants reflected one signal, although it is noteworthy that the effect size of rs2900180 was reduced to 0.99 and the effect size of rs7021880 increased slightly. Additionally, the fine-mapping analyses were performed when conditioning on the strongest associating variant rs7021880. No variants were statistically significant associated with radiographic progression independent of rs7021880 (Additional file 4). In meta-analysis of the ACPA-negative patients of the EAC, Umeå, HCSC-RAC and NDB cohorts rs7021880 was significantly associated with radiographic progression (*P* fixed effects model = 6.35 × 10^−8^) (Additional file 5).

### Downstream effect

To identify functional downstream effects of the region of rs7021880, a database and literature search of transcription studies was performed. The RegulomeDB indicated that this locus has multiple signs of transcriptional activity [21]. Based on RNA expression evaluated by eQTL mapping of peripheral blood samples of 8,086 individuals [22], the minor allele of rs7021880 was negatively correlated with RNA expression of different genes in this region (*cis*-eQTL), with the lowest *P*-value for the expression of *TRAF1* (*P* = 4.93 × 10^−35^) (Additional file 6). However, the strongest correlation between variants in this region with *TRAF1* expression was observed for rs2416804 in *TRAF1* (D’ = 1.000, R^2^ = 0.668 with rs7021880). A study evaluating CD4+ T-cells and monocytes of 461 individuals observed that several variants in the region of rs7021880 had *cis*-eQTL effects on *TRAF1 (*in T-cells) and on *C5* (in monocytes) [23]*.* Both studies explored constitutive expression [22,23]. The effect of a regulatory variant on gene expression, however, may depend on the presence of certain stimuli. Response eQTLs have been studied in different cell types using different stimuli [24-26]. In lymphoblast cell lines of 40 Asian individuals variants in the *C5-TRAF1* region were associated with *TRAF1* expression after phorbol myristate actetate (PMA) stimulation, but not with *C5* expression [24]. Monocytes are cardinal innate immune cells that upon stimulation, exhibit large scale gene transcription and cytokine production. A recent study in 432 individuals showed that the expression of *TRAF1* and *C5* significantly changed in monocytes after stimulation with lipopolysaccharide (LPS) [25]. Furthermore, rs7021880, associated with radiographic progression in our study, as well as several other variants in this region, affected gene expression after two hours of LPS stimulation; a strong *cis*-effect was seen for expression of *TRAF1* (*P* for rs7021880 = 1.20 × 10^−6^, t-stat = −5.00) (Additional file 7) [25]. Similarly, in a comparable study of stimulation-specific eQTLs in dendritic cells derived from peripheral blood monocytes of 534 individuals, the RA-susceptible variant rs881375 in the intergenic *TRAF1-PHF19* region (R^2^ = 0.902, D’ = 1.000 with rs7021880) showed response eQTL after LPS and influenza stimulation (respectively *P* = 6.33 × 10^−8^ and 1.04 × 10^−10^) [26]. Together these data indicate a response eQTL effect on monocytes and dendritic cells derived from monocytes for rs7021880 and its proxy SNPs.

## Discussion

This study aimed to increase the understanding of the relevance of four previously identified risk factors for the severity of joint destruction in RA. To this end, 2,493 RA patients (and 5,895 sets of radiographs) included in six independent cohorts from different parts of Europe and North America were studied. In contrast to previous observations in smaller studies, the variants in *IL-6*, *IL-10* and *FCRL3* were not associated with radiographic progression. This indicates that these variants do not mediate the severity of structural damage in RA. A statistically significant association, confined to the ACPA-negative subgroup of RA was found for rs2900180 in *C5-TRAF1*. Fine-mapping of this region revealed the lowest *P*-value for rs7021880 in *TRAF1,* although this signal was not independent of other variants in this region. The putative relevance of rs7021880 and its surrounding region was supported by differences in RNA expression of *TRAF1* in peripheral blood and monocytes in relation to these genotypes.

We have strongly considered whether the findings on the *C5-TRAF1* region may be false positive. However, despite the fact that all replication cohorts individually had less power than our first cohort and it was unlikely to find statistically significant results in the individual replication cohorts, the obtained effect sizes and directionalities were similar in the Umeå, HCSC-RAC and NDB cohorts. Also, the meta-analysis on these cohorts was highly significant (*P* = 6.35 × 10^−8^ for rs7021880). Therefore, in our view, it is more likely that the finding on *C5-TRAF1* in ACPA-negative RA is a true positive finding than a false positive finding.

The association of rs2900180 in *C5-TRAF1* with radiographic progression was previously observed in two studies on the total RA population [8,9]. The first study concerned 761 RA patients of the Norfolk Arthritis Register (NOAR) with a radiograph after one or five years; 61% of these patients were ACPA-negative. In ACPA-stratified analyses after one year follow-up the effect was significant in ACPA-negative RA-patients but not in ACPA-positive RA patients. The second study reevaluated the NOAR with longitudinal radiographs and also included patients of the Early Rheumatoid Arthritis Study (ERAS); ACPA status was not reported here [9]. The difference in findings in the UK cohorts and our cohorts with regard to ACPA might be the consequence of different frequencies of ACPA-negativity between the cohorts. In the NOAR, the proportion of ACPA-negative patients was higher than in our cohorts (61% versus 28% of the total study population of the six cohorts) [8]. Therefore, the ACPA-negative patients may have contributed more to the results obtained for the NOAR total RA population than in the total RA populations studied here. The consistency of the directionality of the effect (the minor allele associated with more severe damage) in the cohorts studied in the present study and the previously studied cohorts of NOAR and ERAS supported the validity of our findings.

Fine-mapping was performed to explore the *C5-TRAF1* region comprising rs2900180 which is located on chromosome 9 and intergenic between *C5* and *TRAF1*. The threshold for significance of the fine-mapping analyses was corrected for 424 tests which is quite restrictive as the markers included in the analyses are not independent. However, this threshold decreased the chance of false positive findings and, in total, 43 variants had a *P*-value below this threshold. These variants were all highly correlated and located in a 66 Kb region covering *TRAF1* and downstream extending to the *C5-TRAF1* intergenic region and upstream to the *TRAF1- PHF19* intergenic region. Also, these 66 Kb are located within a larger region with a high LD spanning from *C5* to *PHF19* (based on Ceu HapMap data and as described previously [28]). In a conditional analysis including both the initial (rs2900180) and the strongest associating variant (rs7021880), we could not distinguish which variant is the most important. Therefore, the conclusion is that the region encompassing rs7021880 and rs2900180 is associated with radiographic progression. Larger fine-mapping studies are required to conclude definitely on the extent of the region that presumably contains the causal variant.

Previously, we reported that rs10818488, which is also located in the *C5-TRAF1* region (intergenic *C5-TRAF1*), was not associated with radiographic progression in 2,666 RA patients belonging to seven cohorts. No stratification for ACPA was done for this analysis [29]. Also, when rs18018488 was analyzed in the total RA population of the present study which included four cohorts that were studied previously and two additional cohorts, no significant associations were obtained (data not shown). In the present study, rs10818488 was also included in the fine-mapping data of the ACPA-negative patients and did not pass the threshold for multiple testing (which was *P* <1.18 × 10^−4^; the *P*-value for rs10818488 was 6.21 × 10^−4^) (Figure 3C). To explore the relation between rs10818488 and rs2900180 genotypes, the genotypes were compared (Table 2), showing incomplete correlations which is in line with the R^2^ of 0.668 between these two variants. For instance, all patients with genotype AA for rs2900180 had genotype AA for rs10818488, but also other patients had genotype AA for rs18018488. In total, 41 of the 276 ACPA-negative patients (14.9%) had different genotypes which explains the difference in the obtained *P*-values for rs10818488 and rs2900180. The minor allele of rs2900180 that associated in the present study with a higher rate of joint destruction in ACPA-negative RA is also associated with a higher risk of RA [18,28]. Rs2900180 was observed to associate with susceptibility to RA in both Caucasian and Korean patients, in contrast to rs10818488, which was observed to be a risk factor for RA only in Caucasian patients [30]. Hence, apparently not only the association with RA-severity but also the associations with RA-susceptibility are slightly different for rs2900180 and rs10818488.

**Table 2:** **Genotypes of rs10818488 and rs2900180 in the ACPA-negative Leiden EAC patients**

		**rs2900180 (A)**			**Total**
		**GG**	**AG**	**AA**	
rs10818488 (A)	GG	88	0	0	88
	AG	22	113	0	135
	AA	2	17	34	53
Total		112	130	34	276

Presented are the frequencies of the genotypes for rs2900180 (A) and rs10818488 (A) in the 276 ACPA-negative RA patients of the Leiden EAC. The R^2^ between these variants was 0.668. Minor allele frequencies within this group were 35.9% and 43.7% for rs2900180 and rs10818488, respectively. ACPA, anti-citrullinated peptide antibodies; EAC, Early Arthritis Clinic.

A correlation of rs7021880 located in *TRAF1* with *TRAF1* expression was observed in whole blood, although rs7021880 was not the strongest associating genetic variant with *TRAF1* expression [22]. These data are valuable but reflect on a mixture of cells and constitutive expression. Interestingly, very recently two studies evaluated eQTL effects on RNA expression of monocytes or dendritic cells derived from monocytes after several stimuli (response eQTL). These data are attractive since monocytes play a relevant role in the development and progression of RA and because it is conceivable to suggest that variants that associate with progression of the disease are expressed in response to inflammatory stimuli. Hence, differences in such expression may affect the disease course. The expression of *TRAF1* in monocytes was significantly altered after LPS-stimulation compared to naïve monocytes and rs7021880 genotypes were associated with this change in expression after stimulation [25]. Genetic variants might thus affect the level of *TRAF1* expression in response to stimulation. Similar findings were observed in dendritic cells derived from monocytes for rs881375, a good proxy of rs7021880 (R^2^ = 0.902) [26]. *TRAF1* is involved in the NF-ĸB pathway, providing a potential pathway for how these genetic variants may influence progression of joint destruction. The analyses on the large bioinformatics databases supported the notion that the region surrounding rs7021880 has a regulatory function in monocytes, but these databases did not allow us to perform conditional analyses on the genetic variants in this region in relation to RNA expression to identify independent effects. In addition, in several of the studies explored the directionality of the effects on expression was not clearly presented, hampering the interpretation of the potential effects of rs7021880. Although most studies reported on the expression of *TRAF1*, eQTL effects on *C5* in certain cell types have also been reported. The data available do not allow us to conclude whether effects on expression are consistent across cell types. More studies are needed to explain how the *C5-TRAF1* region is relevant for radiographic progression in ACPA-negative RA.

The variants in *IL-6*, *IL-10* and *FCRL3* were not associated with radiographic progression. Also, the directions of the effects between the different cohorts were diverse and no tendency for association was observed. The initial findings on these variants were obtained in studies with a lower number of patients and radiographs than in present study.

This study was started in response to findings of a recent review of the literature on genetic variants that are associated with radiographic progression in RA [22]. Although genotyping data of five of the cohorts were retrieved from the Immunochip, we did not intend to analyze the whole Immunochip, as this was done recently in a study that included three of the six studies that are examined in the present study [20]. This study was focused on the variants for which existing data were contradictory and we went into detail by also performing analyses stratified for ACPA. The total number of genetic variants for radiographic progression that are identified and either replicated in independent cohort studies or found significant in meta-analysis is now thirteen (including *C5-TRAF1*) of which nine were identified in the total RA population (summarized in Table 3). The ACPA-negative subgroup was studied separately in only five studies (excluding the present study). Of these, rs8192916 in *GRZB* and rs1485305 in *OPG* were reported to have a statistically significant association with radiographic progression within the ACPA-negative subgroup; for the other three variants (rs1119132 in *IL4R*, rs11908352 in *MMP-9* and rs451066 on chromosome 14) similarity in effect sizes were reported but statistical significance was not obtained which may be due to smaller sample sizes. The data in Table 3 and the present data show that genetic risk factors for radiographic progression in ACPA-positive and ACPA-negative RA are not similar and further support the notion of two separate disease subsets of RA.

**Table 3:** **Overview of genetic variants for radiographic progression that are replicated in independent cohorts or found significant in meta-analysis**

**Severity variant (risk allele)**	**Located in/nearby gene(s) (chromosome)**	**Risk population**	**Functional associations**
SE [31]	*HLA-DRB1* (chr 6)	All RA	Associated with ACPA-presence
rs4810485 (T) [32]	*CD40* (chr 20)	ACPA-pos	NA
rs7667746 (G) [33]	*IL-15* (chr 4)	All RA^a^	NA
rs7665842 (G) [33]			
rs4371699 (A) [33]			
rs6821171 (A) [33]			
rs1896368 (G) [34]	*DKK-1* (chr 10)	All RA	Serum level DKK-1
rs1896367 (G) [34]			
rs1528873 (A) [34]			
rs2104286 (T) [35]	*IL2RA* (chr 10)	All RA	Serum level IL2Rα
rs8192916 (A) [36]	*GRZB* (chr 14)	All RA^b^	RNA expression in whole blood (eQTL)
rs1119132 (A) [37]	*IL4R* (chr 16)	All RA^c^	NA
rs7607479 (T) [38]	*SPAG16* (chr 2)	ACPA-pos	Serum level MMP-3
rs26232 (C) [39]	*C5orf30* (chr 5)	All RA^d^	NA
rs11908352 (A) [20]	*MMP-9* (chr 20)	All RA^e^	Serum level MMP-9
rs451066 (A) [20]	rs1465788 (chr 14)	All RA^f^	NA
rs1485305 (T) [40]	*OPG* (chr 8)	All RA^g^	NA
rs2900180 (A)	*C5-TRAF1* (chr 9)	ACPA-neg	RNA expression whole blood and monocytes

^a^After adjustment for ACPA, comparable effect sizes were observed (data not shown); ^b^after stratification for ACPA, significant associations were observed in both subgroups (ACPA-negative beta = 1.05 and *P* = 1.98 × 10^−3^; ACPA-positive beta = 1.03 and *P* = 5.40 × 10^−2^); ^c^after stratification for ACPA, comparable effect sizes were observed in both subgroups (data not shown); ^d^after adjustment for ACPA and RF a significant association was observed (beta = 0.90, *P* = 0.03); ^e^after stratification for ACPA, the effect size was larger in the ACPA-positive than in the ACPA-negative subgroup. However, considering the small number of patients per subgroup, none of the analyses resulted in significant *P*-values; ^f^after stratification for ACPA, almost similar effect sizes were observed in both subgroups. However, considering the small number of patients per subgroup, none of the analyses resulted in significant *P*-values; ^g^after stratification, a significant association was observed in ACPA-negative patients (beta = 1.29, *P* = 0.001) but not in ACPA-positive patients, although a similar trend was observed (beta = 1.14, *P* = 0.11). After adjustment for ACPA and RF the association remained significant (beta = 1.20, *P* = 0.02). ACPA, anti-citrullinated peptide antibodies; eQTL, expression quantitative trait locus; MMP, matrix metalloproteinase; NA, not applicable; RA, rheumatoid arthritis.

## Conclusions

In conclusion, in contrast to initial reports, variants in *IL-6, IL-10* and *FCRL3* are not associated with radiographic progression. The association between rs2900180 in *C5-TRAF1* and radiographic progression is confined to ACPA-negative RA. A region surrounding rs2900180 affects *TRAF1* expression in whole blood and monocytes. Further functional studies are needed to elucidate the underlying biological mechanisms in more detail.

## Additional files

Additional file 1:
**Distribution of residuals of the used models for each dataset individually.** Presented are histograms of the residuals for the analyses of radiographic progression for each dataset individually. Radiographic scores for all datasets were log-transformed. For the analyses in the cohorts with multiple radiographs per patient (Leiden EAC, Umeå, Madrid and Wichita) a multivariate normal regression analysis was used. For the datasets with one radiograph per patient (NARAC and NDB), the estimated yearly progression rate was studied by linear regression analysis. All residuals approximate a normal distribution, indicating an appropriate fit of the models. Additional file 2:
**Genetic variants in** ***IL-6*** **(A),** ***IL-10*** **(B),** ***C5-TRAF1*** **(C) and** ***FCRL3*** **(D) in relation to radiographic joint damage progression in ACPA-negative and ACPA-positive subgroups.** Yearly radiographic progression rates per individual cohort and the meta-analyses evaluating the cohorts with ACPA-negative and ACPA-positive patients separately. Analysis on the ACPA-negative subgroup of the Wichita cohort was not performed as it included only three ACPA-negative patients. Presented are the fixed effect *P*-values. Rs1800795 (*IL-6*) ACPA-negative: I^2^ 0.0%, *P* = 0.91; *P* fixed effect = 0.63, *P* random effect = 0.63. Rs1800795 (*IL-6*) ACPA-positive: I^2^ 0.0%, *P* = 0.53; *P* fixed effect = 0.60. *P* random effect = 0.60. Rs1800896 (*IL-10*) ACPA-negative: I^2^ 4.3%, *P* = 0.37; *P* fixed effect = 0.99, *P* random effect = 0.88. Rs1800896 (*IL-10*) ACPA-positive: I^2^ 0.0%, *P* = 0.48; *P* fixed effect = 0.99, *P* random effect = 0.99. Rs2900180 (*C5-TRAF1*) ACPA-negative: I^2^ 33.0%, *P* = 0.22; *P* fixed effect = 5.85 × 10^−7^, *P* random effect = 0.0024. Rs2900180 (*C5-TRAF1*) ACPA-positive: I^2^ 0.0%, *P* = 0.54; *P* fixed effect = 0.13, *P* random effect = 0.13. Rs7528684 (*FRCL3*) ACPA-negative: I^2^ 40.4%. *P* = 0.17; *P* fixed effect = 0.91, *P* random effect = 0.56. Rs7528684 (*FRCL3*) ACPA-positive: I^2^ 7.4%. *P* = 0.37; *P* fixed effect = 0.91, *P* random effect = 0.95. Additional file 3:
**Results of the association with radiographic progression of fine-mapped data from Chr 9:122,656 Kb-122,927Kb in ACPA-negative Leiden EAC-patients.**
Additional file 4:
**Fine-mapping analysis of 423 variants in the ACPA-negative patients of the EAC conditioned on rs7021880.** Presented are the *P*-values of the multivariate normal regression analyses of 423 variants in the *C5-TRAF1* region in the ACPA-negative patients of the EAC when conditioned on the strongest associating variant (rs7021880). Using the Bonferroni correction (considering 423 variants studied) the cut-off for statistical significance was set at 1.18 × 10^−4^ as represented by the horizontal line. Additional file 5:
**Rs7021880 in** ***TRAF1*** **in relation to radiographic joint damage progression in ACPA-negative RA patients.** Fine-mapping of the *C5*-*TRAF1* region within the ACPA-negative patients of the Leiden EAC revealed the lowest *P*-value for rs7021880 (*TRAF1*) (beta = 1.05, *P* = 1.39 × 10^−6^). Presented are the yearly radiographic progression rates for genotype rs7021880 per individual cohort and the meta-analysis evaluating all cohorts with ACPA-negative patients. Analysis on the ACPA-negative subgroup of the Wichita cohort was not performed as it included only three ACPA-negative patients. Presented are the fixed effect *P*-values. I^2^ 22.9%, *P* = 0.27; *P* fixed effect = 6.35 × 10^−8^, *P* random effect = 0.0012. Additional file 6:
**Significant correlations of rs7021880 with RNA** ***cis*** **-expression Quantitative Trait Loci (eQTL) in peripheral blood in Westra** ***et al*** **. [** 22 **].** These data have been derived from the blood eQTL browser that accompanies the manuscript of Westra *et al*. [22]. Expression Quantitative Trait Loci (eQTLs) were deemed *cis*-eQTLS when the distance between the SNP position and the probe midpoint was less than 250 Kb, whereas eQTLs with a distance greater than 5 Mb were defined as *trans*-eQTLS. No *trans*-eQTLs were reported for rs7021880. eQTL association tests were performed using a Spearman’s rank correlation. Correlations were converted to Z-scores. If the Z-score is negative, the minor allele is negatively correlated with expression. Correction for multiple testing was performed by controlling the ‘probe-level’ false discovery rate at 0.05 permuting the gene expression data 10 times resulting in significance thresholds for *cis*-eQTLs of 1.31 × 10^−4^ and for trans-eQTLs of 5.10 × 10^−7^ [22]. Additional file 7:
**Significant associations of rs7021880 with RNA** ***cis*** **-expression Quantitative Trait Loci (eQTL) in monocytes after two hours stimulation with lipopolysaccharide in Fairfax** ***et al.*** **[** 25 **].** These data have been derived from Additional file 6 of the article of Fairfax *et al*. [25] *Cis-* and *trans-*acting expression Quantitative Trait Loci (eQTLs) were defined as SNPs showing association with gene expression that were located respectively within a 1 Mb and outside a 1 Mb region of the associated probe. No *trans*-eQTLs were reported for rs7021880. If the t-stat is negative, the minor allele is negatively correlated with expression. Correction for multiple testing was performed by controlling the false discovery rate at 0.05 [25].

## Abbreviations

ACPA: anti-citrullinated peptide antibodies
ACR: American College of Rheumatology
CI: confidence interval
DMARD: disease-modifying antirheumatic drug
EAC: Early Arthritis Clinic
eQTL: expression quantitative trait locus
FCRL3: Fc-receptor-like-3
HCSC-RAC: Hospital Clinico San Carlos – rheumatoid arthritis cohort
HWE: Hardy-Weinberg equilibrium
ICC: intraclass correlation coefficient
IL: interleukin
LD: linking disequilibrium
LPS: lipopolysaccharide
MAF: minor allele frequency
NARAC: North American Rheumatoid Arthritis Consortium
NDB: National Data Bank for Rheumatic Diseases
NSAID: non-steroidal anti-inflammatory drug
PMA: phorbol myristate acetate
RA: rheumatoid arthritis
SHS: Sharp-van der heijde score
SNP: single nucleotide polymorphism
