# Supplementary material for: A genetic study on C5-TRAF1 and progression of joint damage in rheumatoid arthritis
Source: Arthritis Res Ther. 2015 Jan 8;17(1):1. doi: 10.1186/s13075-014-0514-0 (PMC4318544; doi:10.1186/s13075-014-0514-0)
Supplement: Additional file 1: — Distribution of residuals of the used models for each dataset individually. Presented are histograms of the residuals for the analyses of radiographic progression for each dataset individually. Radiographic scores for all datasets were log-transformed. For the analyses in the cohorts with multiple radiographs per patient (Leiden EAC, Umeå, Madrid and Wichita) a multivariate normal regression analysis was used. For the datasets with one radiograph per patient (NARAC and NDB), the estimated yearly progression rate was studied by linear regression analysis. All residuals approximate a normal distribution, indicating an appropriate fit of the models. [file 13075_2014_514_MOESM1_ESM.pdf]

Additional file 1. Distribution of residuals of the used models for each dataset individually

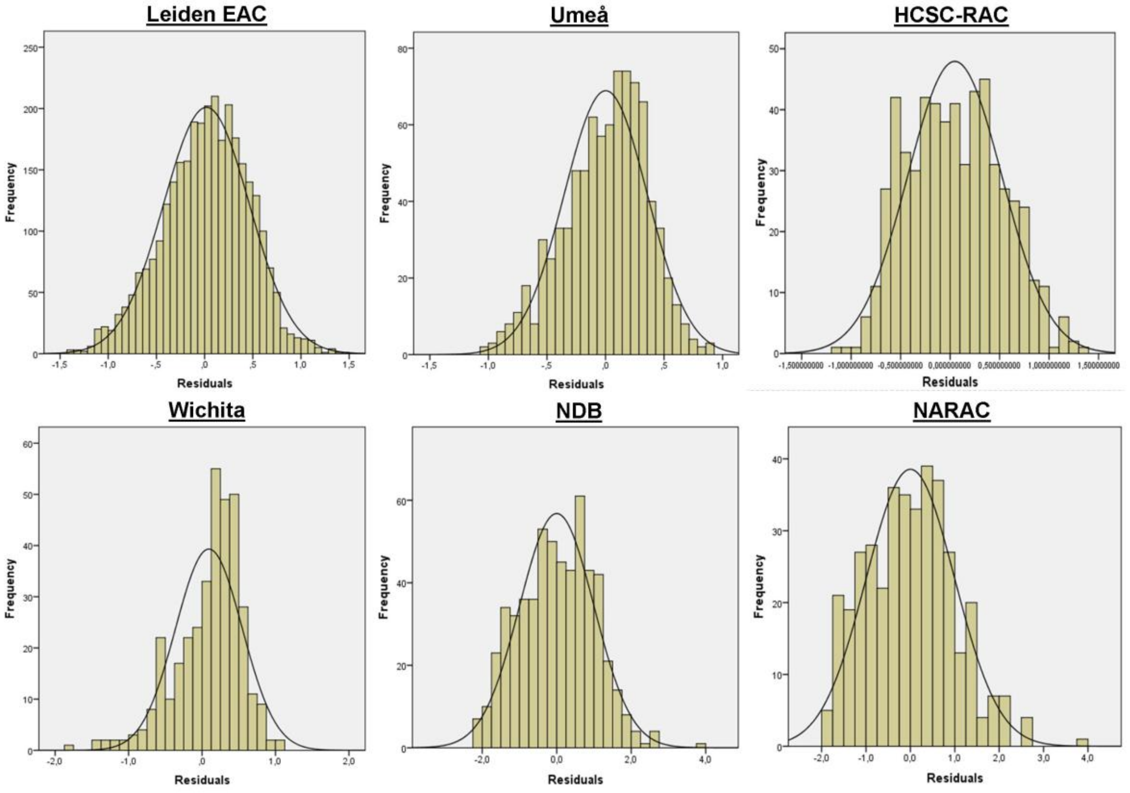

EAC, Early Arthritis Clinic; HCSC-RAC, Hospital Clinico San Carlos – Rheumatoid Arthritis Cohort; NARAC, North American Rheumatoid Arthritis Consortium; NDB, National Data Bank for Rheumatic Diseases
