# Supplementary material for: A genetic study on C5-TRAF1 and progression of joint damage in rheumatoid arthritis
Source: Arthritis Res Ther. 2015 Jan 8;17(1):1. doi: 10.1186/s13075-014-0514-0 (PMC4318544; doi:10.1186/s13075-014-0514-0)

Additional file 2. Genetic variants in *IL-6* (A), *IL-10* (B), *C5-TRAF1* (C) and *FCRL3* (D) in relation to radiographic joint damage progression in ACPA-negative and ACPA-positive subgroups

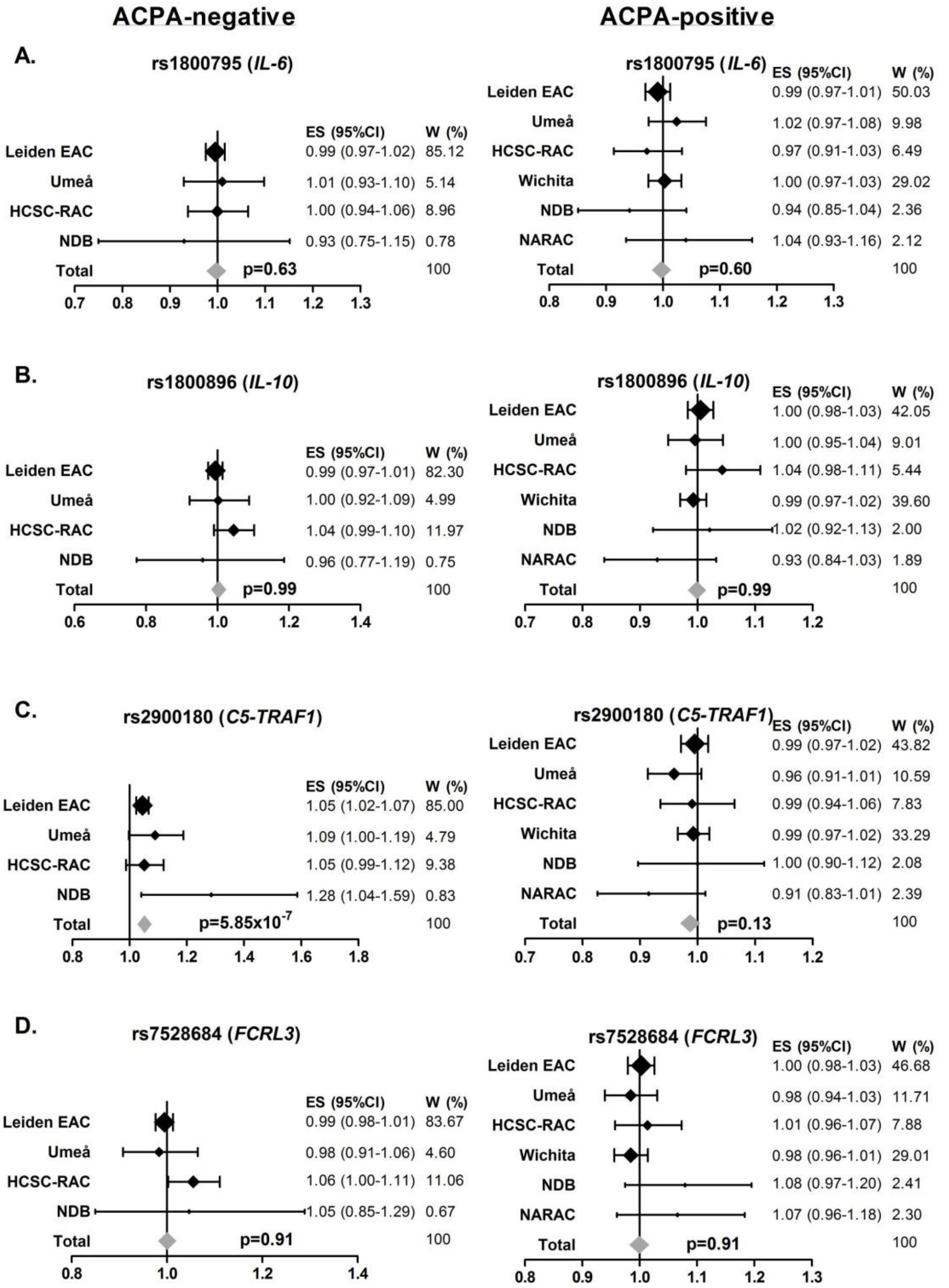

Supplement: Additional file 2: — Genetic variants in IL-6 (A), IL-10 (B), C5-TRAF1 (C) and FCRL3 (D) in relation to radiographic joint damage progression in ACPA-negative and ACPA-positive subgroups. Yearly radiographic progression rates per individual cohort and the meta-analyses evaluating the cohorts with ACPA-negative and ACPA-positive patients separately. Analysis on the ACPA-negative subgroup of the Wichita cohort was not performed as it included only three ACPA-negative patients. Presented are the fixed effect P-values. Rs1800795 (IL-6) ACPA-negative: I2 0.0%, P = 0.91; P fixed effect = 0.63, P random effect = 0.63. Rs1800795 (IL-6) ACPA-positive: I2 0.0%, P = 0.53; P fixed effect = 0.60. P random effect = 0.60. Rs1800896 (IL-10) ACPA-negative: I2 4.3%, P = 0.37; P fixed effect = 0.99, P random effect = 0.88. Rs1800896 (IL-10) ACPA-positive: I2 0.0%, P = 0.48; P fixed effect = 0.99, P random effect = 0.99. Rs2900180 (C5-TRAF1) ACPA-negative: I2 33.0%, P = 0.22; P fixed effect = 5.85 × 10^−7^, P random effect = 0.0024. Rs2900180 (C5-TRAF1) ACPA-positive: I2 0.0%, P = 0.54; P fixed effect = 0.13, P random effect = 0.13. Rs7528684 (FRCL3) ACPA-negative: I2 40.4%. P = 0.17; P fixed effect = 0.91, P random effect = 0.56. Rs7528684 (FRCL3) ACPA-positive: I2 7.4%. P = 0.37; P fixed effect = 0.91, P random effect = 0.95. [file 13075_2014_514_MOESM2_ESM.pdf]
