# Supplementary material for: A genetic study on C5-TRAF1 and progression of joint damage in rheumatoid arthritis
Source: Arthritis Res Ther. 2015 Jan 8;17(1):1. doi: 10.1186/s13075-014-0514-0 (PMC4318544; doi:10.1186/s13075-014-0514-0)
Supplement: Additional file 3: — Results of the association with radiographic progression of fine-mapped data from Chr 9:122,656 Kb-122,927Kb in ACPA-negative Leiden EAC-patients. [file 13075_2014_514_MOESM3_ESM.pdf]

Additional file 3. Results of the association with radiographic progression of fine-mapped data from Chr 9:122,656 Kb-122,927Kb in ACPA-negative Leiden EAC-patients

Immunochip ID	Position	MAF	p-value
imm_9_122656241	122656241	0.152	0.17576083
imm_9_122656537	122656537	0.005	0.133748909
imm_9_122656679	122656679	0.163	0.038283452
imm_9_122658050	122658050	0.270	0.893086684
imm_9_122658792	122658792	0.239	0.900004334
imm_9_122661750	122661750	0.037	0.201372002
imm_9_122662067	122662067	0.160	0.034528384
imm_9_122663593	122663593	0.042	0.39991652
imm_9_122663762	122663762	0.008	0.067346024
imm_9_122664839	122664839	0.018	0.764348107
imm_9_122664970	122664970	0.129	0.159435525
imm_9_122665757	122665757	0.078	0.054809794
imm_9_122666354	122666354	0.123	0.113464202
imm_9_122666891	122666891	0.123	0.113464202
imm_9_122668043	122668043	0.027	0.787172324
imm_9_122668648	122668648	0.120	0.26487516
imm_9_122669339	122669339	0.005	0.025530854
imm_9_122669471	122669471	0.123	0.113464202
imm_9_122670675	122670675	0.078	0.054809794
imm_9_122671382	122671382	0.030	0.143313092
imm_9_122671866	122671866	0.326	0.51105757
imm_9_122672253	122672253	0.079	0.103436801
imm_9_122672650	122672650	0.326	0.51105757
imm_9_122672662	122672662	0.078	0.062156805
imm_9_122673397	122673397	0.007	0.7334476
imm_9_122673769	122673769	0.325	0.51105757
imm_9_122673850	122673850	0.025	0.007197556
imm_9_122674165	122674165	0.038	0.002760377
imm_9_122675942	122675942	0.442	0.000375509
imm_9_122676196	122676196	0.011	0.498784877
imm_9_122676232	122676232	0.094	0.012795175
imm_9_122678090	122678090	0.006	0.271125945
imm_9_122679085	122679085	0.442	0.000510666
imm_9_122680051	122680051	0.447	0.000404815
imm_9_122680083	122680083	0.005	0.980280717
imm_9_122680321	122680321	0.361	1.80775E-05
imm_9_122680989	122680989	0.080	0.268560302
imm_9_122681190	122681190	0.361	1.80775E-05
imm_9_122681404	122681404	0.079	0.268560302
imm_9_122681437	122681437	0.361	1.80775E-05
imm_9_122682172	122682172	0.361	1.80775E-05
imm_9_122683676	122683676	0.446	0.000392189
imm_9_122685556	122685556	0.080	0.268560302
imm_9_122686282	122686282	0.080	0.268560302
imm_9_122687027	122687027	0.046	0.830934011
imm_9_122687906	122687906	0.440	0.000608446
imm_9_122688539	122688539	0.075	0.713913238
imm_9_122690803	122690803	0.364	5.39835E-05
imm_9_122691122	122691122	0.449	0.00092587

imm_9_122692201	122692201	0.364	5.39835E-05
imm_9_122693515	122693515	0.095	0.012795175
imm_9_122694791	122694791	0.080	0.268560302
imm_9_122694905	122694905	0.020	0.705861103
imm_9_122696148	122696148	0.361	2.84678E-05
imm_9_122697323	122697323	0.361	2.84678E-05
imm_9_122698460	122698460	0.361	2.84678E-05
imm_9_122699125	122699125	0.361	2.84678E-05
imm_9_122699160	122699160	0.361	2.84678E-05
imm_9_122700219	122700219	0.361	2.84678E-05
imm_9_122701015	122701015	0.361	2.78344E-05
imm_9_122702439	122702439	0.446	0.000578731
imm_9_122703969	122703969	0.018	0.100713912
imm_9_122704039	122704039	0.030	0.913558122
imm_9_122704416	122704416	0.020	0.380918643
imm_9_122704611	122704611	0.446	0.000578731
imm_9_122704840	122704840	0.446	0.000578731
imm_9_122705118	122705118	0.078	0.117629379
imm_9_122705722	122705722	0.446	0.000578731
imm_9_122706598	122706598	0.368	1.55973E-05
imm_9_122706675	122706675	0.005	0.980280717
imm_9_122706844	122706844	0.006	0.001115303
imm_9_122707615	122707615	0.032	0.22122342
imm_9_122707637	122707637	0.361	2.84678E-05
imm_9_122707854	122707854	0.361	2.84678E-05
imm_9_122708124	122708124	0.005	0.980280717
imm_9_122708147	122708147	0.361	2.84678E-05
imm_9_122710310	122710310	0.005	0.980280717
imm_9_122711341	122711341	0.362	1.67076E-05
imm_9_122711580	122711580	0.005	0.980280717
imm_9_122711658	122711658	0.361	2.84678E-05
imm_9_122711759	122711759	0.008	0.312062868
imm_9_122712204	122712204	0.073	0.112337941
imm_9_122712310	122712310	0.013	0.7743233
imm_9_122713711	122713711	0.338	1.3866E-06
imm_9_122713737	122713737	0.005	0.980280717
imm_9_122713826	122713826	0.005	0.980280717
imm_9_122714954	122714954	0.007	0.323192875
imm_9_122715622	122715622	0.006	0.271125945
imm_9_122716217	122716217	0.443	0.001020534
imm_9_122716303	122716303	0.361	2.84678E-05
imm_9_122716439	122716439	0.006	0.271125945
imm_9_122716520	122716520	0.362	2.84678E-05
imm_9_122716923	122716923	0.361	2.84678E-05
imm_9_122717648	122717648	0.362	2.84678E-05
imm_9_122717950	122717950	0.367	4.40087E-06
imm_9_122718850	122718850	0.029	0.549428318
imm_9_122718906	122718906	0.032	0.00759315
imm_9_122719161	122719161	0.018	0.225438866
imm_9_122719889	122719889	0.446	0.000578731
imm_9_122720821	122720821	0.001	0.019725415
imm_9_122720937	122720937	0.361	2.67672E-05
imm_9_122721076	122721076	0.362	4.34389E-05
imm_9_122721272	122721272	0.072	0.414712072
imm_9_122721431	122721431	0.366	0.000123168

imm_9_122721751	122721751	0.005	0.001130918
imm_9_122722293	122722293	0.079	0.268560302
imm_9_122722842	122722842	0.001	NA*
imm_9_122722927	122722927	0.361	0.001591606
imm_9_122723390	122723390	0.441	0.000608446
imm_9_122723544	122723544	0.172	0.002860122
imm_9_122723655	122723655	0.079	0.268560302
imm_9_122723978	122723978	0.362	2.84678E-05
imm_9_122724016	122724016	0.005	0.980280717
imm_9_122724764	122724764	0.362	2.84678E-05
imm_9_122724913	122724913	0.441	0.000608446
imm_9_122725282	122725282	0.073	0.112337941
imm_9_122726040	122726040	0.361	2.84678E-05
imm_9_122726120	122726120	0.004	0.104345109
imm_9_122727032	122727032	0.362	2.84678E-05
imm_9_122727052	122727052	0.007	0.323192875
imm_9_122727193	122727193	0.362	2.84678E-05
imm_9_122727334	122727334	0.075	0.104357489
imm_9_122727547	122727547	0.005	0.492954678
imm_9_122727655	122727655	0.362	2.84678E-05
imm_9_122727726	122727726	0.441	0.000608446
imm_9_122727847	122727847	0.073	0.112337941
imm_9_122728038	122728038	0.073	0.112638517
imm_9_122728820	122728820	0.014	0.000642961
imm_9_122729418	122729418	0.441	0.000608446
imm_9_122729849	122729849	0.008	0.600932302
imm_9_122730060	122730060	0.441	0.000608446
imm_9_122730113	122730113	0.362	2.84678E-05
imm_9_122730141	122730141	0.000	NA*
imm_9_122730403	122730403	0.073	0.112337941
imm_9_122730778	122730778	0.394	1.19767E-05
imm_9_122730962	122730962	0.364	2.59685E-05
imm_9_122731641	122731641	0.036	0.303486815
imm_9_122732689	122732689	0.363	2.88331E-05
imm_9_122732761	122732761	0.440	0.001253565
imm_9_122734241	122734241	0.006	0.271125945
imm_9_122734609	122734609	0.363	2.88331E-05
imm_9_122734763	122734763	0.363	2.88331E-05
imm_9_122735103	122735103	0.364	4.99004E-06
imm_9_122735471	122735471	0.073	0.112337941
imm_9_122735679	122735679	0.079	0.268560302
imm_9_122736146	122736146	0.448	0.000590267
imm_9_122736577	122736577	0.013	0.115287099
imm_9_122736692	122736692	0.444	0.000621286
imm_9_122737851	122737851	0.007	0.323192875
imm_9_122738010	122738010	0.007	0.806665869
imm_9_122738016	122738016	0.005	0.980280717
imm_9_122738153	122738153	0.073	0.112337941
imm_9_122738311	122738311	0.007	0.323192875
imm_9_122738501	122738501	0.162	0.00739737
imm_9_122738904	122738904	0.007	0.323192875
imm_9_122739730	122739730	0.005	0.980280717
imm_9_122740004	122740004	0.448	0.000590267
imm_9_122740159	122740159	0.072	0.112337941
imm_9_122740317	122740317	0.073	0.112337941

imm_9_122740600	122740600	0.363	2.88331E-05
imm_9_122741705	122741705	0.010	0.739185935
imm_9_122741811	122741811	0.449	0.000590267
imm_9_122741983	122741983	0.079	0.268560302
imm_9_122742159	122742159	0.073	0.112337941
imm_9_122742460	122742460	0.073	0.112337941
imm_9_122743040	122743040	0.007	0.323192875
imm_9_122743507	122743507	0.072	0.579057187
imm_9_122743715	122743715	0.006	0.271125945
imm_9_122744908	122744908	0.442	0.000621286
imm_9_122745110	122745110	0.005	0.980280717
imm_9_122745129	122745129	0.079	0.268560302
imm_9_122745671	122745671	0.079	0.268560302
imm_9_122745766	122745766	0.364	2.88331E-05
imm_9_122746203	122746203	0.363	2.88331E-05
imm_9_122746637	122746637	0.079	0.268560302
imm_9_122747318	122747318	0.448	0.000590267
imm_9_122748094	122748094	0.073	0.112337941
imm_9_122748485	122748485	0.006	0.271125945
imm_9_122749407	122749407	0.006	0.980280717
imm_9_122749962	122749962	0.012	0.291052804
imm_9_122749983	122749983	0.006	0.271125945
imm_9_122752982	122752982	0.006	0.271125945
imm_9_122753153	122753153	0.006	0.980280717
imm_9_122753244	122753244	0.017	0.10923897
imm_9_122754988	122754988	0.006	0.499271857
imm_9_122755317	122755317	0.452	0.002020299
imm_9_122755772	122755772	0.004	0.00477809
imm_9_122755924	122755924	0.076	0.027291927
imm_9_122756370	122756370	0.001	0.594793719
imm_9_122756661	122756661	0.088	0.092968654
imm_9_122757210	122757210	0.088	0.092968654
imm_9_122757661	122757661	0.088	0.092968654
imm_9_122759156	122759156	0.087	0.18020493
imm_9_122760361	122760361	0.006	0.980280717
imm_9_122760709	122760709	0.088	0.092968654
imm_9_122761969	122761969	0.270	0.019400274
imm_9_122762016	122762016	0.076	0.027291927
imm_9_122762317	122762317	0.004	0.469787608
imm_9_122762871	122762871	0.075	0.020383874
imm_9_122763172	122763172	0.452	0.002283815
imm_9_122763278	122763278	0.021	0.228095442
imm_9_122763355	122763355	0.076	0.027291927
imm_9_122763432	122763432	0.006	0.982421017
imm_9_122764329	122764329	0.088	0.092968654
imm_9_122765747	122765747	0.075	0.027291927
imm_9_122765792	122765792	0.006	0.271125945
imm_9_122765850	122765850	0.076	0.027291927
imm_9_122765966	122765966	0.006	0.271125945
imm_9_122767261	122767261	0.076	0.027291927
imm_9_122767354	122767354	0.094	0.005029521
imm_9_122767815	122767815	0.088	0.092968654
imm_9_122768371	122768371	0.007	0.323192875
imm_9_122768434	122768434	0.088	0.092968654
imm_9_122768779	122768779	0.006	0.271125945

imm_9_122769717	122769717	0.076	0.027291927
imm_9_122769783	122769783	0.081	0.029226528
imm_9_122770121	122770121	0.081	0.029226528
imm_9_122770389	122770389	0.006	0.980280717
imm_9_122770510	122770510	0.006	0.271125945
imm_9_122770639	122770639	0.000	NA*
imm_9_122771229	122771229	0.083	0.064033215
imm_9_122772954	122772954	0.209	0.30563038
imm_9_122776839	122776839	0.106	0.000202816
imm_9_122776861	122776861	0.209	0.284684646
imm_9_122777541	122777541	0.106	0.000202816
imm_9_122778184	122778184	0.030	0.861059359
imm_9_122779814	122779814	0.395	0.082845457
imm_9_122780305	122780305	0.396	0.083746707
imm_9_122781643	122781643	0.077	0.352433234
imm_9_122782639	122782639	0.398	0.391127933
imm_9_122783742	122783742	0.103	0.028788358
imm_9_122784073	122784073	0.104	0.028788358
imm_9_122785637	122785637	0.103	0.024378126
imm_9_122786076	122786076	0.394	0.538785876
imm_9_122786259	122786259	0.397	0.148631907
imm_9_122786617	122786617	0.103	0.028788358
imm_9_122787156	122787156	0.106	0.000202816
imm_9_122787762	122787762	0.398	0.558602624
imm_9_122788576	122788576	0.106	0.000202816
imm_9_122788651	122788651	0.105	0.032044289
imm_9_122791621	122791621	0.499	0.392896333
imm_9_122791792	122791792	0.004	0.000739673
imm_9_122792064	122792064	0.000	NA*
imm_9_122793074	122793074	0.104	0.01681525
imm_9_122793421	122793421	0.104	0.01681525
imm_9_122794598	122794598	0.021	0.578919005
imm_9_122795204	122795204	0.104	0.01681525
imm_9_122795397	122795397	0.014	0.000642961
imm_9_122795821	122795821	0.024	0.773570026
imm_9_122795981	122795981	0.476	0.051446073
imm_9_122796786	122796786	0.041	0.004892709
imm_9_122796804	122796804	0.104	0.01681525
imm_9_122797008	122797008	0.476	0.051446073
imm_9_122797233	122797233	0.476	0.051446073
imm_9_122797441	122797441	0.104	0.01681525
imm_9_122798246	122798246	0.420	0.659055297
imm_9_122798453	122798453	0.104	0.01681525
imm_9_122798865	122798865	0.421	0.564430045
imm_9_122799073	122799073	0.476	0.051446073
imm_9_122803731	122803731	0.014	0.000642961
imm_9_122804015	122804015	0.395	0.467705737
imm_9_122804812	122804812	0.104	0.01822553
imm_9_122806347	122806347	0.104	0.032685602
imm_9_122806610	122806610	0.395	0.514432849
imm_9_122809021	122809021	0.421	0.627311186
imm_9_122809663	122809663	0.395	0.514432849
imm_9_122810257	122810257	0.404	0.319668474
imm_9_122811314	122811314	0.118	0.031319684
imm_9_122811620	122811620	0.119	0.031319684

imm_9_122814426	122814426	0.119	0.031319684
imm_9_122814710	122814710	0.119	0.031319684
imm_9_122815581	122815581	0.382	0.57236351
imm_9_122816211	122816211	0.119	0.031319684
imm_9_122817969	122817969	0.499	0.404376496
imm_9_122818509	122818509	0.119	0.031319684
imm_9_122819826	122819826	0.421	0.596852941
imm_9_122821150	122821150	0.119	0.031319684
imm_9_122821256	122821256	0.499	0.404376496
imm_9_122821468	122821468	0.119	0.031319684
imm_9_122821584	122821584	0.119	0.031319684
imm_9_122821591	122821591	0.499	0.404376496
imm_9_122821959	122821959	0.382	0.57236351
imm_9_122822120	122822120	0.119	0.031319684
imm_9_122822877	122822877	0.500	0.404376496
imm_9_122823755	122823755	0.200	0.925238046
imm_9_122823814	122823814	0.201	0.925238046
imm_9_122824169	122824169	0.080	0.012668414
imm_9_122824319	122824319	0.420	0.622467109
imm_9_122824746	122824746	0.049	0.043565689
imm_9_122825021	122825021	0.200	0.925238046
imm_9_122825366	122825366	0.200	0.925238046
imm_9_122825559	122825559	0.003	0.318909994
imm_9_122825856	122825856	0.200	0.925238046
imm_9_122826096	122826096	0.200	0.925238046
imm_9_122826474	122826474	0.007	0.531070533
imm_9_122826811	122826811	0.019	0.440595374
imm_9_122826912	122826912	0.081	0.012668414
imm_9_122827820	122827820	0.101	0.042641115
imm_9_122828213	122828213	0.228	0.003977529
imm_9_122828384	122828384	0.228	0.003977529
imm_9_122829455	122829455	0.420	0.622467109
imm_9_122830953	122830953	0.228	0.003977529
imm_9_122831826	122831826	0.428	0.017830082
imm_9_122832290	122832290	0.119	0.031319684
imm_9_122832727	122832727	0.199	0.925238046
imm_9_122832810	122832810	0.119	0.031319684
imm_9_122834577	122834577	0.077	0.031871461
imm_9_122835515	122835515	0.228	0.003977529
imm_9_122835667	122835667	0.025	0.000854909
imm_9_122835860	122835860	0.152	0.009459355
imm_9_122837145	122837145	0.228	0.003977529
imm_9_122837364	122837364	0.228	0.003977529
imm_9_122837512	122837512	0.228	0.003977529
imm_9_122838711	122838711	0.428	0.017830082
imm_9_122839336	122839336	0.014	0.754712977
imm_9_122839915	122839915	0.419	0.662231992
imm_9_122840039	122840039	0.081	0.014137139
imm_9_122844597	122844597	0.153	0.009459355
imm_9_122847359	122847359	0.003	0.472396107
imm_9_122848041	122848041	0.228	0.003977529
imm_9_122848784	122848784	0.408	0.728228271
imm_9_122848925	122848925	0.229	0.003977529
imm_9_122849360	122849360	0.229	0.003977529
imm_9_122849558	122849558	0.229	0.003977529

imm_9_122849711	122849711	0.429	0.017830082
imm_9_122849896	122849896	0.002	0.547706758
imm_9_122850268	122850268	0.098	0.047659035
imm_9_122850704	122850704	0.428	0.017830082
imm_9_122850804	122850804	0.098	0.047659035
imm_9_122851538	122851538	0.080	0.012668414
imm_9_122853834	122853834	0.037	0.367013044
imm_9_122854165	122854165	0.080	0.169353923
imm_9_122854996	122854996	0.081	0.012668414
imm_9_122855555	122855555	0.081	0.012668414
imm_9_122855917	122855917	0.418	0.649026623
imm_9_122857103	122857103	0.046	0.025272472
imm_9_122857434	122857434	0.230	0.004430085
imm_9_122858348	122858348	0.001	NA*
imm_9_122859225	122859225	0.076	0.019013325
imm_9_122860134	122860134	0.119	0.031319684
imm_9_122860221	122860221	0.119	0.031319684
imm_9_122860313	122860313	0.228	0.003977529
imm_9_122862268	122862268	0.228	0.003977529
imm_9_122862767	122862767	0.228	0.003977529
imm_9_122863453	122863453	0.229	0.003977529
imm_9_122863745	122863745	0.078	0.012668414
imm_9_122863842	122863842	0.082	0.009714536
imm_9_122864670	122864670	0.228	0.003977529
imm_9_122865005	122865005	0.028	0.190946339
imm_9_122865602	122865602	0.081	0.012668414
imm_9_122866136	122866136	0.429	0.022120939
imm_9_122868038	122868038	0.119	0.031319684
imm_9_122872940	122872940	0.006	0.064596814
imm_9_122874121	122874121	0.022	0.147463196
imm_9_122875020	122875020	0.154	0.016831185
imm_9_122875375	122875375	0.419	0.527118815
imm_9_122875520	122875520	0.154	0.016831185
imm_9_122875525	122875525	0.119	0.031319684
imm_9_122875606	122875606	0.001	0.850511306
imm_9_122875905	122875905	0.120	0.031319684
imm_9_122876474	122876474	0.411	0.627530257
imm_9_122877723	122877723	0.419	0.527118815
imm_9_122878959	122878959	0.148	0.014655051
imm_9_122878978	122878978	0.319	0.000674073
imm_9_122879595	122879595	0.007	0.62058079
imm_9_122881893	122881893	0.313	0.002059477
imm_9_122882537	122882537	0.012	0.266487205
imm_9_122882694	122882694	0.307	0.002503031
imm_9_122883917	122883917	0.419	0.707965958
imm_9_122886404	122886404	0.420	0.731176539
imm_9_122886441	122886441	0.306	0.002503031
imm_9_122887602	122887602	0.007	0.62058079
imm_9_122887781	122887781	0.027	0.234591463
imm_9_122887979	122887979	0.028	0.190946339
imm_9_122888657	122888657	0.413	0.734834309
imm_9_122888658	122888658	0.155	0.001726294
imm_9_122889993	122889993	0.014	0.882848351
imm_9_122890591	122890591	0.320	0.000149065
imm_9_122892486	122892486	0.010	0.381307524

imm_9_122892680	122892680	0.440	0.026873717
imm_9_122896746	122896746	0.014	0.754712977
imm_9_122896906	122896906	0.412	0.734834309
imm_9_122898015	122898015	0.320	0.000149065
imm_9_122898251	122898251	0.313	0.000185914
imm_9_122899486	122899486	0.120	0.02663557
imm_9_122900196	122900196	0.413	0.734834309
imm_9_122900510	122900510	0.413	0.738296235
imm_9_122907291	122907291	0.412	0.765157168
imm_9_122907407	122907407	0.002	0.384959671
imm_9_122907603	122907603	0.313	0.000185914
imm_9_122907642	122907642	0.006	0.510360955
imm_9_122907729	122907729	0.027	0.234591463
imm_9_122908170	122908170	0.320	0.000149065
imm_9_122909336	122909336	0.313	0.000185914
imm_9_122909657	122909657	0.007	0.62058079
imm_9_122909710	122909710	0.077	0.014770264
imm_9_122910284	122910284	0.320	0.000149907
imm_9_122910502	122910502	0.412	0.765157168
imm_9_122910608	122910608	0.148	0.008174788
imm_9_122910705	122910705	0.313	0.000185914
imm_9_122910759	122910759	0.019	0.447337382
imm_9_122911520	122911520	0.320	0.000149065
imm_9_122911977	122911977	0.440	0.026873717
imm_9_122912124	122912124	0.148	0.008174788
imm_9_122913098	122913098	0.007	0.62058079
imm_9_122913297	122913297	0.081	0.115499178
imm_9_122913474	122913474	0.007	0.62058079
imm_9_122915742	122915742	0.041	0.533403409
imm_9_122916126	122916126	0.439	0.026873717
imm_9_122916272	122916272	0.413	0.765157168
imm_9_122918136	122918136	0.025	0.000851598
imm_9_122918175	122918175	0.312	0.000185914
imm_9_122918668	122918668	0.121	0.009075592
imm_9_122919588	122919588	0.413	0.765157168
imm_9_122920391	122920391	0.120	0.02663557
imm_9_122922855	122922855	0.312	0.000185914
imm_9_122924127	122924127	0.319	0.000149065
imm_9_122925096	122925096	0.312	0.000185914
imm_9_122925157	122925157	0.008	0.200655514
imm_9_122925751	122925751	0.120	0.02663557
imm_9_122925976	122925976	0.024	0.291691892
imm_9_122926098	122926098	0.048	0.022436266
imm_9_122926269	122926269	0.014	0.000642961
imm_9_122926554	122926554	0.319	0.000149065

MAF, minor allele frequency; NA, not applicable.

*Because of low MAF multivariate normal regression analysis on radiographic progression cannot be performed.
