# Supplementary material for: A genetic study on C5-TRAF1 and progression of joint damage in rheumatoid arthritis
Source: Arthritis Res Ther. 2015 Jan 8;17(1):1. doi: 10.1186/s13075-014-0514-0 (PMC4318544; doi:10.1186/s13075-014-0514-0)

Additional file 4. Fine-mapping analysis of 423 variants in the ACPA-negative patients of the EAC conditioned on rs7021880

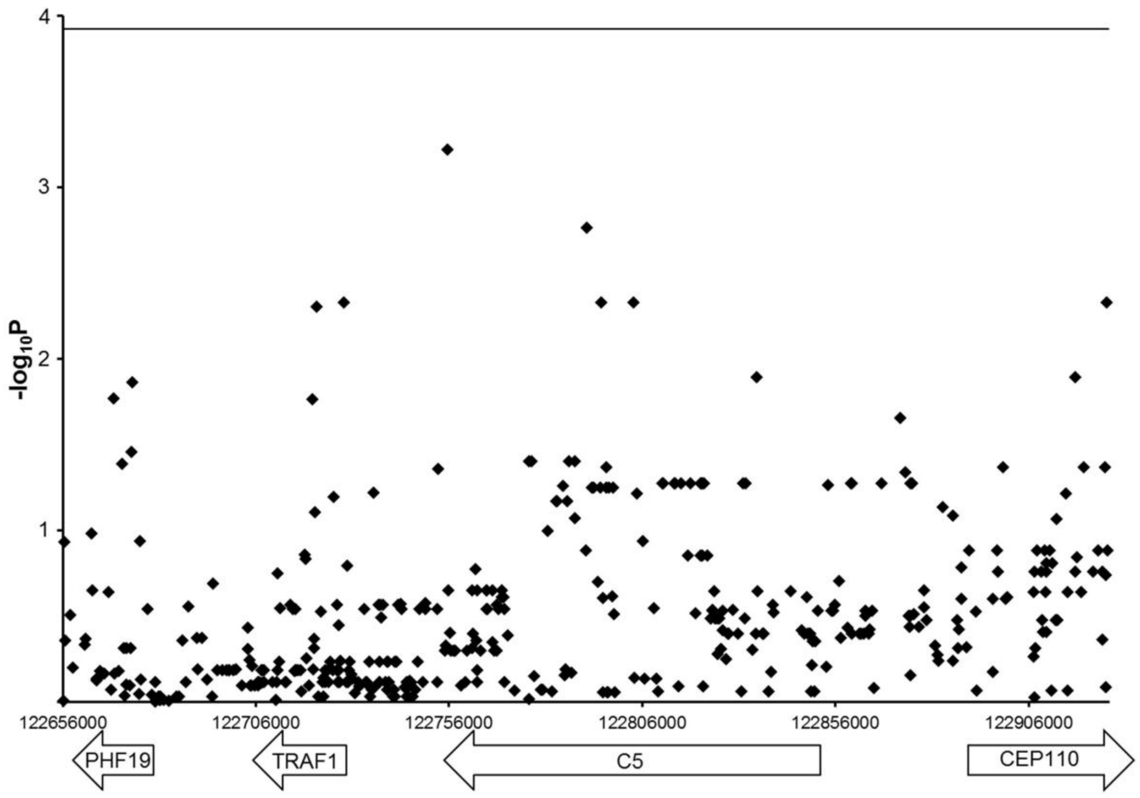

Supplement: Additional file 4: — Fine-mapping analysis of 423 variants in the ACPA-negative patients of the EAC conditioned on rs7021880. Presented are the P-values of the multivariate normal regression analyses of 423 variants in the C5-TRAF1 region in the ACPA-negative patients of the EAC when conditioned on the strongest associating variant (rs7021880). Using the Bonferroni correction (considering 423 variants studied) the cut-off for statistical significance was set at 1.18 × 10^−4^ as represented by the horizontal line. [file 13075_2014_514_MOESM4_ESM.pdf]
