# Supplementary material for: A genetic study on C5-TRAF1 and progression of joint damage in rheumatoid arthritis
Source: Arthritis Res Ther. 2015 Jan 8;17(1):1. doi: 10.1186/s13075-014-0514-0 (PMC4318544; doi:10.1186/s13075-014-0514-0)

Additional file 5. Rs7021880 in *TRAF1* in relation to radiographic joint damage progression in ACPA-negative RA patients

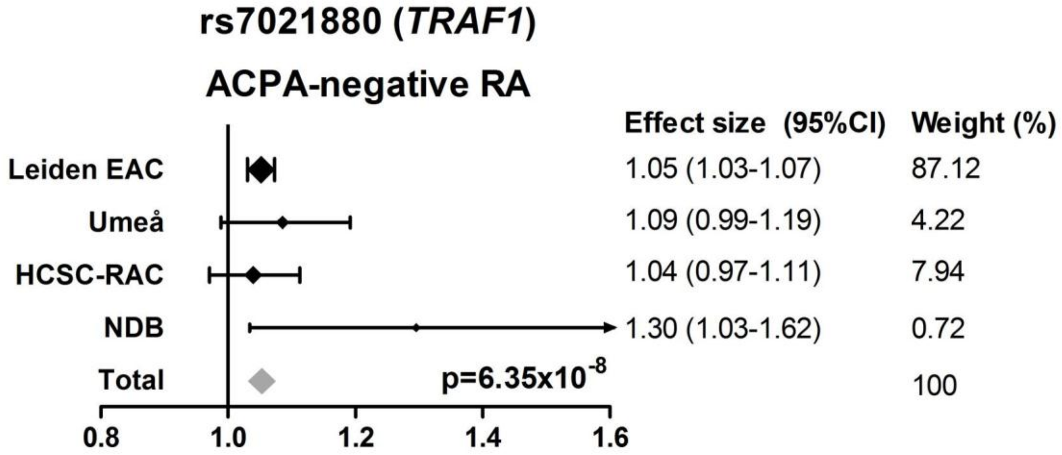

Supplement: Additional file 5: — Rs7021880 in TRAF1 in relation to radiographic joint damage progression in ACPA-negative RA patients. Fine-mapping of the C5-TRAF1 region within the ACPA-negative patients of the Leiden EAC revealed the lowest P-value for rs7021880 (TRAF1) (beta = 1.05, P = 1.39 × 10^−6^). Presented are the yearly radiographic progression rates for genotype rs7021880 per individual cohort and the meta-analysis evaluating all cohorts with ACPA-negative patients. Analysis on the ACPA-negative subgroup of the Wichita cohort was not performed as it included only three ACPA-negative patients. Presented are the fixed effect P-values. I2 22.9%, P = 0.27; P fixed effect = 6.35 × 10^−8^, P random effect = 0.0012. [file 13075_2014_514_MOESM5_ESM.pdf]
