# Supplementary material for: A genetic study on C5-TRAF1 and progression of joint damage in rheumatoid arthritis
Source: Arthritis Res Ther. 2015 Jan 8;17(1):1. doi: 10.1186/s13075-014-0514-0 (PMC4318544; doi:10.1186/s13075-014-0514-0)
Supplement: Additional file 6: — Significant correlations of rs7021880 with RNA cis -expression Quantitative Trait Loci (eQTL) in peripheral blood in Westra et al . [ 22 ]. These data have been derived from the blood eQTL browser that accompanies the manuscript of Westra et al. [22]. Expression Quantitative Trait Loci (eQTLs) were deemed cis-eQTLS when the distance between the SNP position and the probe midpoint was less than 250 Kb, whereas eQTLs with a distance greater than 5 Mb were defined as trans-eQTLS. No trans-eQTLs were reported for rs7021880. eQTL association tests were performed using a Spearman’s rank correlation. Correlations were converted to Z-scores. If the Z-score is negative, the minor allele is negatively correlated with expression. Correction for multiple testing was performed by controlling the ‘probe-level’ false discovery rate at 0.05 permuting the gene expression data 10 times resulting in significance thresholds for cis-eQTLs of 1.31 × 10^−4^ and for trans-eQTLs of 5.10 × 10^−7^ [22]. [file 13075_2014_514_MOESM6_ESM.pdf]

Additional file 6. Significant correlations of rs7021880 with RNA *cis*-expression Quantitative Trait Loci (eQTL) in peripheral blood in Westra et al. (ref)

Expressed gene	Probe mid-point	Minor allele	Z-score	p-value
TRAF1	122705130	C	-12.35	$4.93x10^{-35}$
C5	122754560	C	-11.83	$2.86x10^{-32}$
PHF19	122671898	C	-5.41	$6.46x10^{-8}$
PHF19	122657956	C	-4.72	$2.38x10^{-6}$

These data has been derived from the blood eQTL browser that accompanies the manuscript of Westra et al (see reference).
