# Supplementary material for: A genetic study on C5-TRAF1 and progression of joint damage in rheumatoid arthritis
Source: Arthritis Res Ther. 2015 Jan 8;17(1):1. doi: 10.1186/s13075-014-0514-0 (PMC4318544; doi:10.1186/s13075-014-0514-0)
Supplement: Additional file 7: — Significant associations of rs7021880 with RNA cis -expression Quantitative Trait Loci (eQTL) in monocytes after two hours stimulation with lipopolysaccharide in Fairfax et al. [ 25 ]. These data have been derived from Additional file 6 of the article of Fairfax et al. [25] Cis- and trans-acting expression Quantitative Trait Loci (eQTLs) were defined as SNPs showing association with gene expression that were located respectively within a 1 Mb and outside a 1 Mb region of the associated probe. No trans-eQTLs were reported for rs7021880. If the t-stat is negative, the minor allele is negatively correlated with expression. Correction for multiple testing was performed by controlling the false discovery rate at 0.05 [25]. [file 13075_2014_514_MOESM7_ESM.pdf]

Additional file 7. Significant associations of rs7021880 with RNA *cis*-expression Quantitative Trait Loci (eQTL) in monocytes after 2 hours stimulation with lipopolysaccharide in Fairfax et al. (ref)

Expressed gene	T-stat	p-value	FDR
CEP110	NA	NA	NA
C5	NA	NA	NA
TRAF1	-5.00	1.20×10^{-6}	2.10×10^{-4}
PHF19	NA	NA	NA
PHF19	NA	NA	NA
LOC253039	5.33	2.42×10^{-7}	4.95×10^{-5}
MEGF9	NA	NA	NA
GSN	NA	NA	NA
CDK5RAP2	-2.93	3.74×10^{-3}	0.17

These data has been derived from Supplementary Table 2 of the manuscript of Fairfax et al (see reference).
